# Aprosexia in Children

**Published:** 1891-03-07

**Authors:** 


### THE PRACTITIONER'S MIRROR AND RETROSPECT.

[*The Editor will be glad to receive offers of co-operation and contributions from members of the profession. All letters should be addressed to* THE EDITOR, THE LODGE, PORCHESTER SQUARE, LONDON, W.]

### MEDICINE.

### APROSEXIA IN CHILDREN.

Aprosexia, the Greek for heedlessness, is a term introduced in 1887 by Professor Guye, of Hamburg, for the mental symptoms associated with postnasal growths. Undoubtedly the characteristic dulness and apathy of children who have these adenoid vegetations blocking up the posterior nares, and occluding by pressure the orifices of the eustachian tubes, is increased by the resulting deafness, but it is certainly not entirely due to this condition, which is altogether out of proportion to the degree of deafness found in many of the cases. The children are stunted, ill-nourished, and listless. Professor Guye attributed the mental phenomena to pressure on the lymphatics and veins in the posterior nares and oof of the naso-pharynx, these being in connection with the veins and lymphatics at the base of the prefrontal portion of the brain and its membranes, and Professor Ferrier's experiments with monkeys in which the prefrontal lobes were removed seem to confirm Guye's surmise. The obstruction to nasal respiration itself causes much injury to the health, but when it is considered that these are the least serious consequences of the persistence of these postnasal growths, the necessity for greater attention to this affection should be borne in mind.

